# Differential Associations of Alcohol Use With Ischemic Heart Disease Mortality by Socioeconomic Status in the US, 1997-2018

**DOI:** 10.1001/jamanetworkopen.2023.54270

**Published:** 2024-02-01

**Authors:** Yachen Zhu, Laura Llamosas-Falcón, William Kerr, Klajdi Puka, Charlotte Probst

**Affiliations:** 1 Alcohol Research Group, Public Health Institute, Emeryville, California; 2 Institute for Mental Health Policy Research, Centre for Addiction and Mental Health (CAMH), Toronto, Ontario, Canada; 3 Department of Epidemiology and Biostatistics, Western University, London, Ontario, Canada; 4 Heidelberg Institute of Global Health, Universitätsklinikum Heidelberg, Heidelberg, Germany; 5 Department of Psychiatry, University of Toronto, Toronto, Ontario, Canada

## Abstract

**Importance:**

People with low socioeconomic status (SES) experience greater burden from alcohol-attributable health conditions and mortality at equal levels of alcohol consumption compared with those with high SES. A U-shaped association has been established between alcohol use and ischemic heart disease (IHD), but no study has explored how such an association differs by SES in the US.

**Objective:**

To investigate how the association of alcohol use with ischemic heart disease mortality differs by SES in the general US population.

**Design, Setting, and Participants:**

This cohort study used record-linked, cross-sectional National Health Interview Survey data for US adults aged 25 years and older, covering 1997 to 2018 with mortality follow-up until 2019. Data analysis was performed from March to June 2023.

**Exposures:**

SES (operationalized using education attainment) and alcohol consumption were obtained from self-reported questionnaires.

**Main Outcomes and Measures:**

The outcome was time to IHD mortality or last presumed alive by December 31, 2019. Cox proportional hazard models were applied to evaluate the interaction of SES and alcohol use on IHD mortality, with age as the time scale. Sex-stratified analyses were performed, adjusting for race and ethnicity, marital status, smoking, body mass index, physical activity, and survey year. Fine-Gray subdistribution models were applied to account for competing risks.

**Results:**

This cohort study of 524 035 participants (mean [SD] age at baseline, 50.3 [16.2] years; 290 492 women [51.5%]) found a statistically significantly greater protective association of drinking less than 20 g per day (vs lifetime abstinence) with IHD mortality in the high-SES group compared with the low-SES group (interaction term hazard ratio [HR], 1.22 [95% CI, 1.02-1.45] in men; HR, 1.35 [95% CI, 1.09-1.67] in women). In addition, the differential associations of drinking less than 20 g per day with IHD mortality by SES were observed only among people with less than monthly heavy episodic drinking (HED) (interaction term, HR, 1.20 [95% CI, 1.01-1.43] in men; HR, 1.34 [95% CI, 1.08-1.67] in women); no difference was found in people with at least monthly HED. Among women there was a greater protective association of drinking less than 20 g per day with IHD mortality in the high-SES group than the middle-SES group (interaction term, HR, 1.35 [95% CI, 1.06-1.72]). Among men, the harmful association of drinking more than 60 g per day with IHD mortality in the low-SES group was largely explained by other behavioral risk factors (ie, smoking, body mass index, and physical activity).

**Conclusions and Relevance:**

This cohort study found a greater protective association between drinking less than 20 g per day with less than monthly HED and IHD mortality in the high-SES group compared with the low-SES group, in both sexes even after adjusting for key covariables and behavioral risk factors. The findings suggest that public health interventions on alcohol use should account for different socioeconomic backgrounds when assessing the level of risk related to alcohol exposure, bearing in mind that levels of consumption deemed safe regarding a specific outcome such as IHD may indeed be less safe or not safe across all sociodemographic groups.

## Key Points

**Question:**

Does socioeconomic status (SES) modify the association of alcohol use with ischemic heart disease (IHD) mortality in the general US population?

**Findings:**

This cohort study of 524 035 participants in the US found a more pronounced protective association of light-to-moderate drinking with IHD mortality in the high-SES group in both sexes, even after adjusting for key covariables and behavioral risk factors. Other behavioral risk factors, including smoking, body mass index, and physical activity, largely explained the harmful association between chronic heavy drinking and IHD mortality in men with low SES.

**Meaning:**

Public health interventions on alcohol use should account for different socioeconomic backgrounds when assessing the level of risk related to alcohol exposure, bearing in mind that levels of consumption deemed safe regarding a specific outcome such as IHD may indeed be less safe or not safe across all sociodemographic groups.

## Teaser

This cohort study investigates how the association of alcohol use with ischemic heart disease (IHD) mortality differs by socioeconomic status in the general US population.

## Introduction

Cardiovascular diseases (CVDs) are the leading causes of death in the US.^1,2^ People with low socioeconomic status (SES) bear an excess burden of CVDs, especially ischemic heart disease (IHD).^3,4^ From 2000 to 2010, growing socioeconomic inequalities in US life expectancy were found to be largely attributed to major declines in IHD and ischemic stroke mortality among people with high SES, whereas people with low SES experienced much slower declines in IHD and ischemic stroke mortality rates.^5^

Alcohol use has long been identified as an important health behavior that contributes to socioeconomic inequalities in mortality.^6^ People with low SES experience greater burden from 100% alcohol-attributable health conditions (eg, alcohol poisonings and alcoholic liver cirrhosis) and mortality^7,8,9,10^ at equal or lower levels of alcohol consumption^10,11^ compared with those with high SES.^6,12,13,14,15,16^ This public health phenomenon is often referred to as the *alcohol-harm paradox*.^17,18,19^ Overall, a U-shaped relationship has been established between alcohol use and IHD, in that light-to-moderate drinking with no heavy episodic drinking (HED) showed a protective association with IHD,^20,21,22,23^ whereas HED and higher average levels of alcohol consumption showed a harmful association.^21,24,25^

A meta-analysis^26^ of alcohol consumption and IHD mortality showed evidence of effect modification by sex, cohort age, ethnicity, and whether studies adjusted for heart health, yet without identifying the potential effect modification by SES. Norström et al^27^ investigated the association of alcohol per capita consumption with IHD mortality rates by education group using Swedish quarterly time-series data. They found increases in IHD mortality rates with increasing per capita consumption in the low and middle education groups, and the elevated risk of IHD mortality associated with per capita consumption was significantly higher the lower the education level. The research provided evidence for the socioeconomic gradient in the association of alcohol with IHD mortality at the population level. The only study currently investigating the socioeconomic gradient in the association between alcohol use and CVD mortality at the individual level was performed by Degerud et al^28^ using data from the Norwegian population-based health survey. The study found that the protective association of CVD mortality with moderately frequent alcohol consumption compared with infrequent consumption was more pronounced among participants with higher SES, and that very frequent alcohol consumption (4-7 times per week) was associated with an increased risk of CVD mortality only among participants with low SES.^28^ However, to our knowledge, no study to date has investigated the effect modification of alcohol on IHD mortality by SES in the US.

Given the high prevalence of both alcohol use^29,30^ and IHD^31^ in the US, and noticeable socioeconomic disparities in IHD mortality, with low SES being associated with an increased risk,^4,5^ it is thus imperative to investigate the role of SES in the association of alcohol use with IHD mortality. To this end, we explored the interaction between SES and alcohol use on IHD mortality using a large representative US sample linked to cause-specific mortality data. We hypothesized that the harmful association of heavy drinking with IHD mortality would be higher among people with low SES compared with those with high SES, whereas the protective association of light-to-moderate drinking with IHD mortality would be more pronounced among people with high SES than those with low SES.

## Methods

### Data Source

We extracted baseline demographics and data for health behaviors from the 1997 to 2018 National Health Interview Survey (NHIS). NHIS is an annual, nationally representative, cross-sectional survey that collects health information on the civilian noninstitutionalized population residing in the 50 states and the District of Columbia in the US. It uses a complex multistage probability sampling design to select participants.^32^ All adult participants in the NHIS provided written informed consent. Data collection for NHIS and analysis of restricted-use data were approved by the Ethics Review Board of the National Center for Health Statistics and the US Office of Management and Budget. This study followed the Strengthening the Reporting of Observational Studies in Epidemiology (STROBE) reporting guideline.

### Measures

The outcome, time to IHD death or last presumed alive, was extracted from the 2019 National Death Index and then linked to NHIS data.^33^ IHD mortality was operationalized using the *International Statistical Classification of Diseases and Related Health Problems, Tenth Revision* codes I20 to I25 and *International Classification of Diseases, Ninth Revision* codes 410 to 414. The baseline age was calculated as the difference between the interview date and date of birth of a participant. The end age was calculated as the difference between date of death and date of birth if the participant was deceased by December 31, 2019, and as the difference between December 31, 2019, and date of birth if the participant was still alive on December 31, 2019. IHD mortality, age at NHIS interview, and age at death were restricted-use variables and were accessed through the National Center for Health Statistics Research Data Center. We restricted the sample to participants aged 25 years or older, assuming that by the study enrollment most of the participants had attained their highest level of education.

We operationalized SES using educational attainment: (1) high school degree or less (hereafter, low education), (2) some college (middle education), and (3) bachelor’s degree or more (high education, reference), given that education is less likely to be negatively affected by alcohol use and be subject to reverse causality compared with income and occupation.^13,27,34,35^ Family income level (high income, ≥400% of federal poverty threshold [reference]; middle income, 200%-399% of federal poverty threshold; and low income, <199% of federal poverty threshold) was used as a secondary indicator of SES.

We calculated daily grams of alcohol consumption by the following equation: (the number of days drank alcohol in the past year / 365) × (the average number of drinks on days drank × 14 g/drink), assuming 14 g of pure alcohol per standard drink. Given the known U-shaped relationship with IHD mortality,^20,21,22,23^ we operationalized alcohol intake using categorical drinking status in the past 12 months with the same increment of 20 g per day from 0 g per day to above to facilitate the comparison between sexes. Because men and women have substantially different ranges of alcohol intake and there were very few women drinking more than 40 g per day, we combined all women who drank more than 20 g per day, thus ending up with different number of categories for men and women (ie, 6 groups for men and 4 groups for women): lifetime abstainer (never drank alcohol in the past 12 months and never had ≥12 drinks in any 1 year; reference group), former drinker (never drank alcohol in the past 12 months but ever had ≥12 drinks in any 1 year), category I (past year daily average of >0 to ≤20 g for both women and men), category II (past year daily average of >20 to ≤40 g for men and >20 g for women), category III (past-year daily average of >40 to ≤60 g for men only), and category IV (past-year daily average >60 g for men only). We used lifetime abstainers (rather than current abstainers) as the reference group to reduce the sick-quitter bias, because the inclusion of former drinkers among current abstainers may lead to an overestimation of the protective associations and an underestimation of the harmful associations with alcohol consumption.^21,36,37^ In a sensitivity analysis, we also accounted for occasional HED (ie, had ≥5 drinks at least 1 occasion at least once a month) by further categorizing drinking status into 8 groups: lifetime abstainer, former drinker, category I with less than monthly HED, category I with at least monthly HED, category II with less than monthly HED, category II with at least monthly HED, category III (for men only), and category IV (for men only).

We selected potential confounders a priori on the basis of previous literature^21^ and adjusted for marital status (married or living with partner vs never married, widowed, divorced, or separated), self-identified race and ethnicity (non-Hispanic Black, Hispanic, and other [non-Hispanic Asian and Pacific Islander, American Indian and Alaska Native, and non-Hispanic all other race groups] vs non-Hispanic White), smoking status (former smoker, current someday smoker, current everyday smoker vs never smoker), body mass index (BMI; calculated as weight in kilograms divided by height in meters squared) (underweight, <18.5; healthy weight, 18.5-24.99; overweight, 25-29.99; and obesity, ≥30),^38,39^ physical activity (sedentary, 0 minutes per week; somewhat active, <150 minutes per week; and active, ≥150 minutes per week),^39,40^ and survey year.

### Statistical Analysis

Data analysis was performed from March to June 2023. We applied Cox proportional hazard (PH) models to evaluate the interaction effect of education and alcohol use on IHD mortality. Because age is considered as the most important risk factor for IHD mortality, we used age rather than follow-up years as the time scale in the survival analyses to allow for a complete nonparametric age effect, which provides more accurate risk relationships than using follow-up years and adjusting for age as a covariate.^41,42^ Because sex is known to be an effect modifier on alcohol use and IHD mortality,^21,25,26^ as a first step, we explored the interaction between sex (women vs men) and alcohol use (coded as lifetime abstainer [reference], former drinker, >0 to ≤20 g per day, and >20 g per day for both sexes for this specific analysis) in the overall sample. In our main analyses, we then further conducted stratified analyses by sex to account for different risk relationships for women and men (ie, Cox PH models with interaction between education and alcohol use adjusting for covariates in both sexes). We ran 2 models for the sex-stratified analyses. In model 1 (minimally adjusted model), we adjusted for marital status, race and ethnicity, and survey year. In model 2 (main model), we additionally adjusted for behavioral risk factors including smoking status, BMI, and physical activity. We checked the PH assumption by evaluating the independence between Schoenfeld residuals and age and did not find any evidence for a violation of the PH assumption.^43^ We accounted for the complex survey design of NHIS in the Cox models through survey weights, strata, and primary sampling units. We exponentiated the Cox PH regression estimates to obtain hazard ratios (HRs).

In sensitivity analyses, we first accounted for HED by recategorizing alcohol use to incorporate the status of HED in category I and II drinking levels, given that HED does not provide protective effect on IHD^21^ and that people with low SES are more likely to engage in HED^11^ and are more susceptible to the adverse effects of HED than those with high SES.^44^ Second, we used family income instead of education as an indicator of SES to test the robustness of findings. Third, we ran Fine-Gray subdistribution models to account for competing risks (ie, causes of death other than IHD), which may alter people’s probability of experiencing IHD death.^45^ However, these models cannot adjust for complex survey design and can only use follow-up time rather than age as time scale, which may reduce comparability with the Cox PH models used for the main analyses. We additionally adjusted for categorical age (25-34, 35-44, 45-54, 55-64, 65-74, 75-84, and ≥85 years) in the Fine-Gray models.

Following guidelines on analyses of effect modification and interaction,^46,47^ we presented results in 2 formats: (1) HRs (95% CIs) with a single common reference group for SES and alcohol use combined (ie, lifetime abstainer with high SES), and (2) HRs (95% CIs and *P* values) that measured the effect modification of SES on the association of alcohol use with IHD mortality. All statistical analyses were conducted in R statistical software version 4.2.3 (R Project for Statistical Computing).^48^ A 2-sided *P* < .05 was considered statistically significant.

## Results

Our final data included 524 035 participants (mean [SD] age at baseline, 50.3 [16.2] years; 233 543 men [48.5%] and 290 492 women [51.5%]). Detailed information about missing data is shown in eTable 1 in Supplement 1. Sample characteristics are shown in Table 1. The mean (SD) follow-up time was 10.3 (6.1) years. A total of 13 256 IHD deaths occurred during follow-up, with an overall survey-adjusted mortality rate of 204.8 per 100 000 person-years (95% CI, 199.4-210.3 per 100 000 person-years). The survey-adjusted mortality rates were 247.6 per 100 000 person-years (95% CI, 239.8-255.4 per 100 000 person-years) for men, 165.1 per 100 000 person-years (95% CI, 159.2-170.9 per 100 000 person-years) for women, 102.2 per 100 000 person-years (95% CI, 96.3-108.1 per 100 000 person-years) for the high-education group, 166.8 per 100 000 person-years (95% CI, 159.0-174.5 per 100 000 person-years) for the middle-education group, and 301.0 per 100 000 person-years (95% CI, 291.3-310.6 per 100 000 person-years) for the low-education group.

**Table 1.** Characteristics of Study Participants by Education Level, National Health Interview Survey, 1997-2018

Characteristic	Participants, No. (weighted %)							*P* value
	Overall (N = 524 035)	Men (n = 233 543 [48.5%])			Women (n = 290 492 [51.5%])			
		Bachelors (n = 69 079)	High school (n = 101 999)	Some college (n = 62 465)	Bachelors (n = 76 788)	High school (n = 129 686)	Some college (n = 84 018)	
Duration of follow-up, mean (SD), y	10.3 (6.1)	10.1 (6.1)	10.3 (6.1)	10.2 (6.1)	10.0 (6.1)	10.5 (6.1)	10.4 (6.1)	<.001
Age at baseline, mean (SD), y	50.3 (16.2)	49.0 (15.2)	50.8 (16.3)	48.2 (15.1)	47.2 (14.8)	54.7 (17.5)	49.2 (15.8)	<.001
Alcohol use for all participants, mean (SD), g/d	5.7 (19.0)	7.8 (20.1)	9.0 (29.7)	8.5 (19.5)	4.1 (10.0)	2.4 (12.3)	3.3 (9.7)	<.001
Sample size of current drinkers, No.	325 601	54 062	62 816	45 503	55 584	54 894	52 742	NA
Alcohol use for current drinkers only, mean (SD), g/d	9.0 (23.0)	9.0 (23.0)	14.8 (36.6)	12.2 (24.4)	5.6 (11.2)	5.4 (18.9)	5.2 (12.2)	<.001
Alcohol use^a^								
Lifetime abstainer	160 725 (28.6)	11 454 (16.1)	26 915 (25.9)	11 548 (18.4)	18 096 (22.8)	66 192 (48.7)	26 520 (30.3)	<.001
Former drinker	37 709 (6.8)	3563 (4.8)	12 268 (11.1)	5414 (8.0)	3108 (3.8)	8600 (6.3)	4756 (5.3)	
Category I	287 928 (57.3)	46 321 (68.4)	50 123 (50.7)	37 607 (61.6)	52 503 (69.4)	51 582 (42.3)	49 792 (60.8)	
Category II	26 742 (5.3)	5692 (8.0)	6859 (6.7)	4848 (7.5)	3081 (4.0)	3312 (2.7)	2950 (3.5)	
Category III	5687 (1.1)	1363 (1.9)	2722 (2.6)	1602 (2.5)	0	0	0	
Category IV	5244 (1.0)	686 (0.9)	3112 (3.0)	1446 (2.1)	0	0	0	
Smoking								
Never smoker	292 254 (56.0)	43 860 (65.0)	40 283 (39.9)	28508 (47.0)	55 058 (72.9)	76 565 (57.8)	47 980 (57.6)	<.001
Former smoker	127 703 (24.6)	18 003 (25.7)	31 454 (30.2)	18 966 (30.2)	15 246 (19.3)	24 929 (19.8)	19 105 (22.8)	
Current someday smoker	21 401 (3.8)	2414 (3.2)	5466 (5.1)	3227 (4.8)	2163 (2.6)	4566 (3.3)	3565 (3.9)	
Current everyday smoker	82 677 (15.6)	4802 (6.2)	24 796 (24.8)	11 764 (17.9)	4321 (5.2)	23 626 (19.2)	13 368 (15.7)	
Body mass index^b^								
Healthy weight (18.5-24.99)	181 061 (34.1)	22 368 (31.1)	27 778 (26.1)	15 745 (23.9)	39 116 (52.0)	44 566 (34.8)	31 488 (38.1)	<.001
Underweight (<18.5)	8724 (1.5)	338 (0.4)	986 (0.9)	348 (0.5)	2149 (2.7)	3105 (2.3)	1798 (2.1)	
Overweight (25-29.99)	187 626 (36.3)	31 728 (46.4)	43 255 (42.1)	27 148 (43.7)	20 062 (26.2)	40 868 (31.1)	24 565 (29.1)	
Obese (≥30)	146 624 (28.1)	14 645 (22.0)	29 980 (30.9)	19 224 (31.9)	15 461 (19.1)	41 147 (31.7)	26 167 (30.7)	
Physical activity								
Active (≥150 min/wk)	226 244 (45.0)	44 119 (64.0)	35 614 (35.9)	31 910 (51.5)	43 830 (57.6)	35 210 (28.8)	35 561 (43.5)	<.001
Sedentary (0 min/wk)	202 923 (36.5)	13 439 (18.8)	51 228 (48.9)	20 096 (31.4)	16 941 (21.1)	70 936 (52.4)	30 283 (34.4)	
Somewhat active (<150 min/wk)	94 868 (18.5)	11 521 (17.1)	15 157 (15.2)	10 459 (17.2)	16 017 (21.3)	23 540 (18.8)	18 174 (22.1)	
Race and ethnicity								
White	342 084 (70.7)	52 566 (78.5)	59 656 (64.0)	44 173 (73.8)	56 237 (76.3)	72 952 (64.1)	56 500 (72.8)	<.001
Black	72 601 (11.3)	5266 (6.7)	15 061 (12.4)	8232 (11.5)	8044 (8.5)	21 970 (13.5)	14 028 (13.5)	
Hispanic	81 415 (12.6)	4685 (6.0)	23 706 (20.1)	7308 (10.5)	5813 (6.2)	29 695 (18.0)	10 208 (9.7)	
Other^c^	27 935 (5.5)	6562 (8.9)	3576 (3.5)	2752 (4.2)	6694 (9.0)	5069 (4.3)	3282 (3.9)	
Income, % of federal poverty threshold								
High (≥400)	157 087 (34.7)	39 382 (59.8)	17 862 (20.2)	22 091 (38.3)	40 029 (56.3)	15 710 (16.1)	22 013 (31.1)	<.001
Low (<199)	142 955 (22.4)	5926 (6.9)	36 720 (32.1)	11 650 (16.1)	7706 (8.0)	58 024 (37.8)	22 929 (22.4)	
Middle (200-399)	128 998 (25.0)	12 596 (17.3)	28 487 (28.9)	18 603 (29.5)	16 475 (19.5)	28 502 (25.0)	24 335 (29.2)	
Missing	94 995 (17.9)	11 175 (16.0)	18 930 (18.8)	10 121 (16.1)	12 578 (16.2)	27 450 (21.1)	14 741 (17.4)	
Marital status								
Married or cohabitating	286 008 (67.9)	44 340 (76.5)	60 284 (70.1)	36 578 (70.8)	43 925 (70.7)	59 474 (59.2)	41 407 (63.2)	<.001
Not married or cohabitating	238 027 (32.1)	24 739 (23.5)	41 715 (29.9)	25 887 (29.2)	32 863 (29.3)	70 212 (40.8)	42 611 (36.8)	

Abbreviation: NA, not applicable.

^a^ Alcohol use categories are defined as follows: lifetime abstainer (never drank alcohol in the past 12 months and never had ≥12 drinks in any 1 year; reference group), former drinker (never drank alcohol in the past 12 months but ever had ≥12 drinks in any 1 year), category I (past year daily average of >0 to ≤20 g for both women and men), category II (past year daily average of >20 to ≤40 g for men and >20 g for women), category III (past-year daily average of >40 to ≤60 g for men only), and category IV (past-year daily average >60 g for men only).

^b^ Body mass index is calculated as weight in kilograms divided by height in meters squared.

^c^ Other race and ethnicity included non-Hispanic Asian and Pacific Islander, American Indian and Alaska Native, and non-Hispanic all other race groups.

In the overall sample, we found a significant interaction between sex (women vs men) and drinking (>0 to ≤20 g per day vs lifetime abstainer) with an HR of 0.81 (95% CI, 0.74-0.89), indicating that the protective association between drinking more than 0 to less than or equal to 20 g per day (relative to lifetime abstinence) and IHD mortality was more pronounced in women compared with men. Furthermore, the risk of drinking more than 20 g per day was significantly lower in women than in men (interaction term HR, 0.66; 95% CI, 0.51-0.84). Given these interaction effects, we performed sex-stratified analyses described later.

In the main model (model 2) (Table 2), we found a statistically significant interaction between low education and drinking more than 0 to less than or equal to 20 g per day in both men (HR, 1.22; 95% CI, 1.02-1.45) and women (HR, 1.35; 95% CI, 1.09-1.67), indicating a greater protective association between drinking more than 0 to less than or equal to 20 g per day (vs lifetime abstinence) and IHD mortality in the high-education group compared with the low-education group. In addition, among women we observed a significant interaction between middle education and drinking more than 0 to less than or equal to 20 g per day, with an HR of 1.35 (95% CI, 1.06-1.72), pointing in the same direction. These educational gradients are shown in the Figure on relative IHD mortality hazards compared with lifetime abstainers with high education.

**Table 2.** Adjusted HRs With 95% CIs for the Association of Alcohol Use and Education on Ischemic Heart Disease Mortality Stratified by Sex

Variable^a^	HR (95% CI)			Low vs high education		Middle vs high education	
	High education	Middle education	Low education	HR (95% CI)	*P* value	HR (95% CI)	*P* value
Model 1							
Men							
Lifetime abstainer	1 [Reference]	1.60 (1.34-1.91)^b^	1.50 (1.30-1.74)^b^	1.50 (1.30-1.74)	<.001^b^	1.60 (1.34-1.91)	<.001^b^
Former drinker	1.23 (0.98-1.54)	1.62 (1.34-1.97)^b^	1.82 (1.57-2.10)^b^	0.98 (0.76-1.27)	.89	0.82 (0.61-1.12)	.21
>0 to ≤20 g/d	0.66 (0.56-0.77)^b^	1.10 (0.94-1.28)	1.26 (1.10-1.45)^b^	1.28 (1.07-1.53)	.007^c^	1.05 (0.85-1.29)	.68
>20 to ≤40 g/d	0.74 (0.58-0.95)^d^	0.97 (0.75-1.25)	1.42 (1.16-1.74)^b^	1.28 (0.94-1.73)	.12	0.82 (0.58-1.16)	.26
>40 to ≤60 g/d	1.08 (0.73-1.61)	1.89 (1.38-2.59)^b^	1.43 (1.08-1.91)^d^	0.88 (0.54-1.43)	.61	1.09 (0.65-1.83)	.74
>60 g/d	1.07 (0.63-1.80)	1.53 (1.05-2.22)^d^	2.44 (1.84-3.23)^b^	1.52 (0.85-2.73)	.16	0.90 (0.49-1.65)	.73
Women							
Lifetime abstainer	1 [Reference]	1.34 (1.13-1.59)^b^	1.57 (1.35-1.84)^b^	1.57 (1.35-1.84)	<.001^b^	1.34 (1.13-1.59)	.001^b^
Former drinker	1.28 (0.89-1.82)	1.34 (1.03-1.73)^d^	2.04 (1.70-2.46)^b^	1.02 (0.70-1.48)	.93	0.79 (0.51-1.20)	.26
>0 to ≤20 g/d	0.48 (0.39-0.58)^b^	0.91 (0.76-1.10)	1.08 (0.91-1.28)	1.44 (1.16-1.78)	.001^b^	1.43 (1.13-1.82)	.003^c^
>20 g/d	0.61 (0.37-1.02)	1.03 (0.69-1.53)	1.06 (0.74-1.52)	1.10 (0.60-2.03)	.75	1.26 (0.66-2.40)	.49
Model 2							
Men							
Lifetime abstainer	1 [Reference]	1.37 (1.15-1.64)^b^	1.18 (1.02-1.36)^d^	1.18 (1.02-1.36)	.03^d^	1.37 (1.15-1.64)	.001^b^
Former drinker	1.06 (0.85-1.34)	1.22 (1.00-1.49)^d^	1.28 (1.11-1.49)^b^	1.02 (0.79-1.33)	.85	0.84 (0.62-1.14)	.25
>0 to ≤20 g/d	0.66 (0.57-0.77)^b^	0.92 (0.78-1.07)	0.95 (0.82-1.09)	1.22 (1.02-1.45)	.03^d^	1.01 (0.82-1.25)	.92
>20 to ≤40 g/d	0.70 (0.54-0.90)^c^	0.77 (0.59-1.00)	1.00 (0.81-1.23)	1.22 (0.90-1.66)	.21	0.81 (0.57-1.15)	.23
>40 to ≤60 g/d	0.97 (0.65-1.44)	1.38 (1.01-1.88)^d^	0.94 (0.71-1.26)	0.83 (0.51-1.35)	.44	1.04 (0.62-1.75)	.89
>60 g/d	0.86 (0.51-1.44)	1.05 (0.72-1.52)	1.46 (1.10-1.95)^c^	1.45 (0.82-2.58)	.20	0.89 (0.49-1.62)	.71
Women							
Lifetime abstainer	1 [Reference]	1.23 (1.04-1.47)^d^	1.34 (1.14-1.57)^b^	1.34 (1.14-1.57)	<.001^b^	1.23 (1.04-1.47)	.02^d^
Former drinker	1.13 (0.79-1.60)	1.06 (0.82-1.38)	1.50 (1.24-1.82)^b^	1.00 (0.69-1.45)	.99	0.76 (0.50-1.17)	.21
>0 to ≤20 g/d	0.50 (0.41-0.62)^b^	0.84 (0.70-1.00)	0.91 (0.77-1.08)	1.35 (1.09-1.67)	.006^c^	1.35 (1.06-1.72)	.02^d^
>20 g/d	0.58 (0.34-0.96)^d^	0.81 (0.54-1.21)	0.77 (0.54-1.10)	1.00 (0.54-1.85)	.99	1.14 (0.60-2.20)	.68

Abbreviation: HR, hazard ratio.

^a^ Model 1 was adjusted for marital status, race and ethnicity, and survey year. Model 2 (main model) was additionally adjusted for smoking, body mass index, and physical activity.

^b^ *P* ≤ .001.

^c^ *P* ≤ .01.

^d^ *P* ≤ .05.

**Figure.**
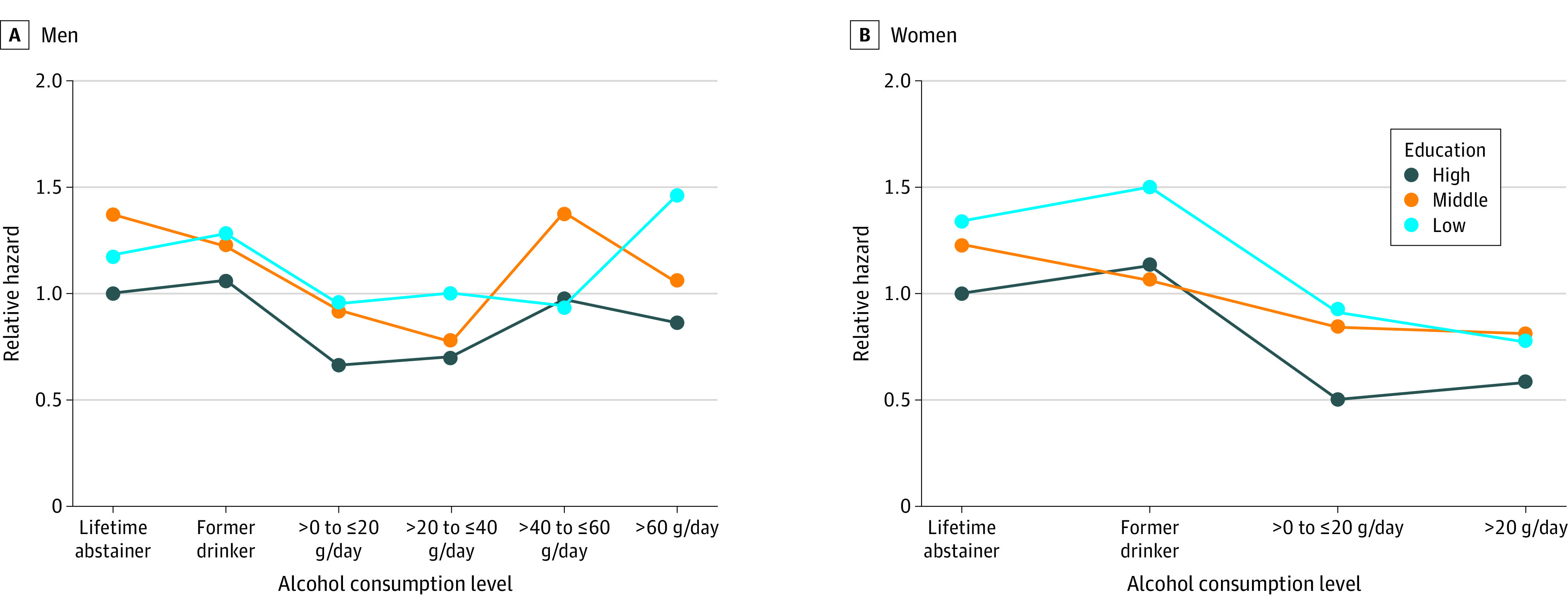
Relative Hazard of Ischemic Heart Disease Mortality Graphs show relative hazards of ischemic heart disease mortality compared with lifetime abstainer with high education, stratifying by sex and adjusting for age (as time scale), marital status, race and ethnicity, smoking status, body mass index, physical activity, and survey year.

In contrast, we did not find a statistically significant interaction between drinking more than 20 g per day and low education. Specifically, we did not observe a detrimental association between drinking more than 20 g per day and IHD mortality in women (Table 2 and eTable 2 in Supplement 1). In the minimally adjusted model, drinking more than 60 g per day showed a harmful association with IHD mortality among men with low education (HR, 1.52; 95% CI, 1.18-1.95) (eTable 2 in Supplement 1). However, this association was largely attenuated (HR, 1.21; 95% CI, 0.94-1.55) (model 2 in eTable 2 in Supplement 1) after further adjusting for other behavioral risk factors (smoking, BMI, and physical activity). Only among those with low education, former drinkers had an elevated risk of IHD mortality among both sexes (men, HR, 1.21 [95% CI, 1.09-1.34]; women, HR, 1.29 [95% CI, 1.14-1.47]) (eTable 2 in Supplement 1). However, after further adjusting for other behavioral risk factors, this association was no longer statistically significant (men, HR, 1.10 [95% CI, 0.99-1.23]; women, HR, 1.13 [95% CI, 0.99-1.29]). Among persons with middle and high education, this elevated risk was not observed.

Accounting for HED, we found a significant interaction between low education and drinking more than 0 to less than or equal to 20 g per day with less than monthly HED among women (HR, 1.34; 95% CI, 1.08-1.67) and men (HR, 1.20; 95% CI, 1.01-1.43), and a significant interaction of middle education with drinking more than 0 to less than or equal to 20 g per day with less than monthly HED among women (HR, 1.36; 95% CI, 1.06-1.73) (eTable 3 in Supplement 1). Yet the interaction of low and middle education with drinking more than 0 to less than or equal to 20 g per day with more than monthly HED was not statistically significant, indicating that the educational gradient in the association of alcohol use with IHD mortality exists only among people with less than monthly HED. Using family income instead of education as the SES indicator and Fine-Gray subdistribution models accounting for competing risks confirmed the results of the main analyses (eTable 4 and eTable 5 in Supplement 1)—that is, a greater protective association with drinking more than 0 to less than or equal to 20 g per day was observed in the high-SES group than in the low-SES group among both sexes.

## Discussion

This cohort study used a unique data source to investigate the heterogeneity in the association of alcohol use with IHD mortality by SES in the US general population. We identified a greater protective association of light-to-moderate drinking (with less than monthly HED) with IHD mortality in the high-SES group than in the low-SES group. To our knowledge, this is the first study that demonstrated such a socioeconomic gradient in the association of alcohol use with IHD mortality in the US.

Degerud et al^28^ found in Norway similar socioeconomic gradient in the protective association of low-to-moderate alcohol use with CVD mortality, and very frequent drinking was associated with higher risk of CVD mortality only among people with low SES. In our study, the harmful association with chronic heavy drinking (>60 g per day)^21^ among low-education men was largely explained by smoking, BMI, and physical activity. Similar to Roerecke et al,^21^ we observed that the protective association with drinking less than 20 g per day was significantly greater in women compared with men. However, we did not observe a higher detrimental association with drinking more than 20 g per day in women than men. Instead, we found a protective association in women, which might be because there were very few women with average alcohol consumption of more than 40 g per day in this study; thus, the association was mainly driven by women who drank more than 20 to less than or equal to 40 g per day.

The poorer protective association of drinking less than 20 g per day with IHD mortality in the low-SES group may, in part, be explained by the fact that people with fewer resources have less access to health care services.^49^ Also, people with low SES are more likely to experience chronic stress across the life course,^50,51^ which is associated with increased blood pressure and the risk of IHD mortality.^52^ Low SES might be associated with other risk factors or unhealthy behaviors that, when combined with alcohol use, counteract the protective effect of low-level drinking and/or exacerbate the negative health effects of heavy drinking. In addition, individuals with higher SES may have healthier behaviors, be more aware of the risks associated with heavy drinking, and have better coping mechanisms or support systems.

Our study has multiple strengths. First, we have a large sample size of 524 035 participants aged 25 years and older enrolled from 1997 to 2018. We accounted for the complex survey design of NHIS, making our results representative of the noninstitutionalized US population. Second, we used a longitudinal design with an average follow-up of 10.3 years, taking into consideration the incubation period of IHD mortality.^53^ Third, we adjusted for important behavioral risk factors, including smoking, physical activity, and BMI, which are likely to cluster among people with low SES.^54^ We found that additionally adjusting for these factors largely explained the harmful association of alcohol use with IHD mortality among former drinkers with low SES, both women and men, as well as the harmful association with drinking greater than 60 g per day in men with low SES. Finally, we used multiple indicators of SES (education and family income) and accounted for competing risks from other causes of death across the life course in sensitivity analyses, which led to similar findings.

### Limitations

This study also has some limitations. First, data on alcohol consumption in the NHIS are based on self-report, which is subject to potential underestimation of the alcohol consumed.^28^ A study using the nationally representative Health Survey for England found that underreporting of alcohol use was more pronounced in people with heavy drinking, yet without differing by SES.^55^ Therefore, the potential underestimation of alcohol consumption is unlikely to invalidate the differential associations by SES. Second, previous studies suggested unhealthy behaviors may cluster in people with low SES.^18^ Although we adjusted for important behavioral risk factors, the possibility of residual confounding remains. For example, we were unable to adjust for dietary habits, although it is assumed that they are closely related to BMI, which was adjusted for in our analyses. Other potential confounders that were uncontrolled include systolic blood pressure, heart rate, triglycerides, diabetes, history of CVD, family history of IHD, drug prescriptions, and urban vs rural areas.^28,56,57,58,59^ In addition, Mendelian randomization suggested that a genetic marker is linked to reduced alcohol consumption and a decreased risk of CVD among light drinkers.^60^ Owing to data limitations, we were unable to account for genetic factors. Third, Ariansen et al^61^ and Stringhini et al^62^ suggested that repeated measurements of risk factors may explain part of the educational gradient in CVD mortality, whereas Franks et al^63^ found that adjusting for time-dependent covariates explained little of the socioeconomic inequalities in IHD risk. However, we only have one measurement for each variable per participant at baseline. In particular, alcohol use could not be accounted for as a time-varying variable, which may lead to an underestimation of its association with IHD mortality with a long follow-up.

## Conclusions

To the best of our knowledge, this cohort study is the first to have analyzed, at the individual level, the socioeconomic gradient in the association of alcohol use with IHD mortality in the US, with a more pronounced protective association with light-to-moderate drinking identified in the high-SES group. This specific protective association should be considered in the context of other known harms from drinking at even low levels, such as cancers, and other studies that do not show an all-cause mortality benefit. Public health interventions should account for different socioeconomic backgrounds in assessing the level of risk related to alcohol exposure, bearing in mind that levels of consumption deemed safe regarding a specific outcome such as IHD may indeed be less safe or not safe across all sociodemographic groups. Future studies are needed to better understand the mechanisms by which alcohol affects cardiovascular health in people with low SES.

## Notes

Supplement 1. **eTable 1.** Missing Data in Key Variables for the Analytical Sample Aged 25 Years and Older **eTable 2.** Adjusted HRs With 95% CIs for the Association Between Alcohol Use and IHD Mortality by Education and Sex **eTable 3.** Adjusted HRs With 95% CIs for the Association of Alcohol Use (With HED Status) and Education on IHD Mortality, Adjusting for Marital Status, Race/Ethnicity, Smoking, BMI, Physical Activity, and Survey Year and Using Age as the Time Scale **eTable 4.** Adjusted HRs With 95% CIs for the Association of Alcohol Use and Family Income on IHD Mortality, Adjusting for Marital Status, Race/Ethnicity, Smoking, BMI, Physical Activity, and Survey Year and Using Age as the Time Scale **eTable 5.** Adjusted HRs With 95% CIs for the Association of Alcohol Use and Education on IHD Mortality From Fine-Gray Subdistribution Models, Adjusting for Baseline Age, Marital Status, Race/Ethnicity, Smoking, BMI, Physical Activity, and Survey Year

Supplement 2.
Data Sharing Statement
