# Supplementary material for: Differential Associations of Alcohol Use With Ischemic Heart Disease Mortality by Socioeconomic Status in the US, 1997-2018
Source: JAMA Netw Open. 2024 Feb 1;7(2):e2354270. doi: 10.1001/jamanetworkopen.2023.54270 (PMC10835511; doi:10.1001/jamanetworkopen.2023.54270)
Supplement: Supplement 1. — eTable 1. Missing Data in Key Variables for the Analytical Sample Aged 25 Years and Older eTable 2. Adjusted HRs With 95% CIs for the Association Between Alcohol Use and IHD Mortality by Education and Sex eTable 3. Adjusted HRs With 95% CIs for the Association of Alcohol Use (With HED Status) and Education on IHD Mortality, Adjusting for Marital Status, Race/Ethnicity, Smoking, BMI, Physical Activity, and Survey Year and Using Age as the Time Scale eTable 4. Adjusted HRs With 95% CIs for the Association of Alcohol Use and Family Income on IHD Mortality, Adjusting for Marital Status, Race/Ethnicity, Smoking, BMI, Physical Activity, and Survey Year and Using Age as the Time Scale eTable 5. Adjusted HRs With 95% CIs for the Association of Alcohol Use and Education on IHD Mortality From Fine-Gray Subdistribution Models, Adjusting for Baseline Age, Marital Status, Race/Ethnicity, Smoking, BMI, Physical Activity, and Survey Year [file jamanetwopen-e2354270-s001.pdf]

Supplemental Online Content

Zhu Y, Llamosas-Falcón L, Kerr W, Puka K, Probst C. Differential associations of alcohol use with ischemic heart disease mortality by socioeconomic status in the US, 1997-2018. *JAMA Netw Open*. 2024;7(2):e2354270. doi:10.1001/jamanetworkopen.2023.54270

eTable 1. Missing Data in Key Variables for the Analytical Sample Aged 25 Years and Older

eTable 2. Adjusted HRs With 95% CIs for the Association Between Alcohol Use and IHD Mortality by Education and Sex

eTable 3. Adjusted HRs With 95% CIs for the Association of Alcohol Use (With HED Status) and Education on IHD Mortality, Adjusting for Marital Status, Race/Ethnicity, Smoking, BMI, Physical Activity, and Survey Year and Using Age as the Time Scale

eTable 4. Adjusted HRs With 95% CIs for the Association of Alcohol Use and Family Income on IHD Mortality, Adjusting for Marital Status, Race/Ethnicity, Smoking, BMI, Physical Activity, and Survey Year and Using Age as the Time Scale

eTable 5. Adjusted HRs With 95% CIs for the Association of Alcohol Use and Education on IHD Mortality From Fine-Gray Subdistribution Models, Adjusting for Baseline Age, Marital Status, Race/Ethnicity, Smoking, BMI, Physical Activity, and Survey Year

This supplemental material has been provided by the authors to give readers additional information about their work.

© 2024 Zhu Y et al. *JAMA Network Open.*

eTable 1. Missing Data in Key Variables for the Analytical Sample Aged 25 Years and Older

	N (%) with missing data
Education	4,764 (0.8%)
Race/ethnicity	18 (0.003%)
Marital status	2,218 (0.4%)
Alcohol use	12,905 (2.2%)
Smoking	4,708 (0.8%)
BMI	23,507 (3.9%)
Physical activity	29,241 (4.9%)
Survey weight	21,139 (3.5%)

© 2024 Zhu Y et al. *JAMA Network Open.*

eTable 2. Adjusted HRs With 95% CIs for the Association Between Alcohol Use and IHD Mortality by Education and Sex

	Education-stratified analyses		
	High education	Middle education	Low education
Model 1			
Men			
Lifetime abstainer	1.00	1.00	1.00
Former drinker	1.19 (0.95-1.50)	1.02 (0.85-1.24)	1.21 (1.09-1.34)***
(0, 20] g/day	0.69 (0.59-0.81)***	0.70 (0.60-0.81)***	0.81 (0.74-0.89)***
(20, 40] g/day	0.77 (0.60-0.99)*	0.62 (0.48-0.80)***	0.90 (0.76-1.07)
(40, 60] g/day	1.08 (0.73-1.61)	1.22 (0.89-1.68)	0.90 (0.69-1.19)
>60 g/day	1.16 (0.68-1.98)	1.00 (0.68-1.46)	1.52 (1.18-1.95)***
Women			
Lifetime abstainer	1.00	1.00	1.00
Former drinker	1.42 (0.98-2.04)	1.01 (0.80-1.27)	1.29 (1.14-1.47)***
(0, 20] g/day	0.59 (0.48-0.72)***	0.70 (0.60-0.81)***	0.66 (0.61-0.73)***
>20 g/day	0.75 (0.44-1.27)	0.78 (0.53-1.14)	0.64 (0.46-0.89)**
Model 2 (main model)			
Men			
Lifetime abstainer	1.00	1.00	1.00
Former drinker	1.08 (0.85-1.37)	0.87 (0.72-1.05)	1.10 (0.99-1.23)
(0, 20] g/day	0.70 (0.60-0.83)***	0.66 (0.57-0.77)***	0.79 (0.72-0.87)***
(20, 40] g/day	0.74 (0.56-0.96)*	0.55 (0.43-0.72)***	0.83 (0.70-0.99)*
(40, 60] g/day	1.00 (0.67-1.51)	1.01 (0.73-1.38)	0.78 (0.59-1.03)
>60 g/day	0.91 (0.54-1.54)	0.78 (0.53-1.15)	1.21 (0.94-1.55)
Women			
Lifetime abstainer	1.00	1.00	1.00
Former drinker	1.32 (0.91-1.91)	0.84 (0.66-1.06)	1.13 (0.99-1.29)
(0, 20] g/day	0.62 (0.49-0.78)***	0.68 (0.58-0.79)***	0.66 (0.61-0.72)***
>20 g/day	0.73 (0.42-1.26)	0.65 (0.44-0.97)*	0.56 (0.41-0.78)***

Note: Model 1 adjusted for marital status, race/ethnicity, and survey year; Model 2 additionally adjusted for smoking, BMI, and physical activity.
*p≤.05
**p≤.01
*** p≤.001

© 2024 Zhu Y et al. *JAMA Network Open.*

eTable 3. Adjusted HRs With 95% CIs for the Association of Alcohol Use (With HED Status) and Education on IHD Mortality, Adjusting for Marital Status, Race/Ethnicity, Smoking, BMI, Physical Activity, and Survey Year and Using Age as the Time Scale

	High education	Middle education	Low education	Low vs. High	Middle vs. High
Men					
Lifetime abstainer	1.00	1.37 (1.15-1.64)***	1.18 (1.02-1.36)*	1.18 (1.02-1.36), p=.03*	1.37 (1.15-1.64), p=.001***
Former drinker	1.07 (0.85-1.34)	1.22 (1.00-1.49)*	1.28 (1.11-1.49)***	1.02 (0.79-1.33), p=.86	0.84 (0.62-1.13), p=.25
(0, 20] g/day with less than monthly HED	0.66 (0.56-0.77)***	0.92 (0.79-1.08)	0.93 (0.81-1.07)	1.20 (1.01-1.43), p=.04*	1.02 (0.83-1.27), p=.84
(0, 20] g/day with at least monthly HED	0.76 (0.43-1.36)	0.83 (0.59-1.17)	1.15 (0.91-1.46)	1.28 (0.70-2.36), p=.42	0.79 (0.41-1.56), p=.50
(20, 40] g/day with less than monthly HED	0.72 (0.54-0.95)*	0.78 (0.56-1.09)	1.07 (0.83-1.39)	1.27 (0.88-1.83), p=.19	0.79 (0.52-1.21), p=.29
(20, 40] g/day with at least monthly HED	0.62 (0.37-1.04)	0.68 (0.47-0.99)*	0.90 (0.67-1.19)	1.24 (0.69-2.22), p=.48	0.81 (0.43-1.54), p=.52
(40, 60] g/day	0.97 (0.65-1.44)	1.38 (1.01-1.89)*	0.94 (0.71-1.26)	0.83 (0.51-1.35), p=.44	1.04 (0.62-1.75), p=.89
>60 g/day	0.86 (0.51-1.44)	1.05 (0.72-1.53)	1.47 (1.11-1.95)**	1.46 (0.82-2.59), p=.20	0.89 (0.49-1.63), p=.71
Women					
Lifetime abstainer	1.00	1.23 (1.04-1.47)*	1.34 (1.14-1.57)***	1.34 (1.14-1.57), p<.001***	1.23 (1.04-1.47), p=.02*
Former drinker	1.13 (0.79-1.60)	1.06 (0.82-1.38)	1.50 (1.24-1.82)***	1.00 (0.69-1.45), p=.99	0.76 (0.50-1.17), p=.21
(0, 20] g/day with less than monthly HED	0.50 (0.41-0.62)***	0.84 (0.70-1.01)	0.91 (0.77-1.08)	1.34 (1.08-1.67), p=.007**	1.36 (1.06-1.73), p=.01*
(0, 20] g/day with at least monthly HED	Suppressed (n<5)	0.37 (0.14-0.96)*	0.89 (0.53-1.52)	1.76 (0.44-7.08), p=.43	0.78 (0.16-3.92), p=.77
>20 g/day with less than monthly HED	0.65 (0.37-1.17)	0.62 (0.39-1.00)*	0.73 (0.48-1.12)	0.84 (0.42-1.67), p=.62	0.77 (0.37-1.61), p=.49
>20 g/day with at least monthly HED	0.38 (0.15-0.98)*	1.33 (0.65-2.71)	0.86 (0.50-1.48)	1.68 (0.58-4.88), p=.34	2.81 (0.86-9.13), p=.09

*p≤.05
**p≤.01
*** p≤.001

© 2024 Zhu Y et al. *JAMA Network Open.*

eTable 4. Adjusted HRs With 95% CIs for the Association of Alcohol Use and Family Income on IHD Mortality, Adjusting for Marital Status, Race/Ethnicity, Smoking, BMI, Physical Activity, and Survey Year and Using Age as the Time Scale

	High income	Middle income	Low income	Low vs. High	Middle vs. High
Men					
Lifetime abstainer	1.00	1.37 (1.16-1.61)***	1.46 (1.24-1.73)***	1.46 (1.24-1.73), p<.001***	1.37 (1.16-1.61), p<.001***
Former drinker	1.00 (0.80-1.26)	1.32 (1.10-1.57)**	1.59 (1.34-1.90)***	1.08 (0.83-1.41), p=.55	0.96 (0.72-1.28), p=.78
(0, 20] g/day	0.70 (0.60-0.83)***	0.94 (0.80-1.11)	1.31 (1.13-1.53)***	1.27 (1.03-1.57), p=.03*	0.98 (0.80-1.20), p=.85
(20, 40] g/day	0.78 (0.61-0.98)*	1.01 (0.79-1.30)	1.29 (0.99-1.67)	1.13 (0.81-1.59), p=.47	0.95 (0.69-1.32), p=.78
(40, 60] g/day	1.03 (0.74-1.45)	1.06 (0.74-1.52)	1.23 (0.83-1.83)	0.82 (0.49-1.37), p=.44	0.75 (0.46-1.22), p=.25
>60 g/day	1.06 (0.69-1.64)	1.61 (1.11-2.35)*	1.46 (1.04-2.04)*	0.94 (0.54-1.62), p=.81	1.11 (0.65-1.89), p=.70
Women					
Lifetime abstainer	1.00	1.38 (1.15-1.65)***	1.63 (1.38-1.92)***	1.63 (1.38-1.92), p<.001***	1.38 (1.15-1.65), p=.001***
Former drinker	1.08 (0.76-1.53)	1.47 (1.12-1.93)**	1.78 (1.43-2.21)***	1.02 (0.69-1.50), p=.94	0.99 (0.65-1.51), p=.97
(0, 20] g/day	0.57 (0.46-0.69)***	0.89 (0.74-1.07)	1.24 (1.03-1.49)*	1.35 (1.08-1.68), p=.008**	1.14 (0.90-1.45), p=.28
>20 g/day	0.62 (0.39-0.99)*	0.91 (0.59-1.42)	0.87 (0.56-1.35)	0.87 (0.46-1.64), p=.66	1.07 (0.57-2.00), p=.82

*p≤.05
**p≤.01
*** p≤.001

© 2024 Zhu Y et al. *JAMA Network Open.*

eTable 5. Adjusted HRs With 95% CIs for the Association of Alcohol Use and Education on IHD Mortality From Fine-Gray Subdistribution Models, Adjusting for Baseline Age, Marital Status, Race/Ethnicity, Smoking, BMI, Physical Activity, and Survey Year

	High education	Middle education	Low education	Low vs. High	Middle vs. High
Men					
Lifetime abstainer	1.00	1.24 (1.07-1.44)**	1.13 (0.99-1.28)	1.13 (0.99-1.28), p=.07	1.24 (1.07-1.44), p=.004**
Former drinker	1.03 (0.84-1.25)	1.15 (0.97-1.36)	1.12 (0.97-1.28)	0.98 (0.79-1.21), p=.83	0.89 (0.69-1.15), p=.36
(0, 20] g/day	0.71 (0.62-0.82)***	0.96 (0.84-1.10)	0.98 (0.86-1.12)	1.25 (1.07-1.47), p=.004**	1.09 (0.91-1.31), p=.33
(20, 40] g/day	0.74 (0.59-0.94)*	0.84 (0.66-1.06)	1.01 (0.84-1.21)	1.24 (0.94-1.63), p=.12	0.92 (0.67-1.26), p=.59
(40, 60] g/day	1.00 (0.71-1.41)	1.33 (0.99-1.78)	0.87 (0.67-1.12)	0.78 (0.51-1.18), p=.23	1.09 (0.70-1.70), p=.70
>60 g/day	0.91 (0.56-1.47)	1.07 (0.77-1.49)	1.19 (0.96-1.49)	1.20 (0.71-2.02), p=.49	0.99 (0.55-1.76), p=.96
Women					
Lifetime abstainer	1.00	1.26 (1.09-1.46)**	1.36 (1.18-1.57)***	1.36 (1.18-1.57), p<.001***	1.26 (1.09-1.46), p=.002**
Former drinker	1.17 (0.86-1.58)	1.15 (0.90-1.45)	1.43 (1.14-1.79)**	0.92 (0.67-1.27), p=.62	0.79 (0.55-1.13), p=.20
(0, 20] g/day	0.62 (0.49-0.79)	0.97 (0.76-1.24)	1.06 (0.86-1.31)	1.26 (1.04-1.53), p=.02*	1.24 (1.00-1.53), p=.05*
>20 g/day	0.68 (0.41-1.12)	1.00 (0.69-1.45)	0.93 (0.68-1.26)	1.01 (0.57-1.81), p=.97	1.17 (0.66-2.07), p=.60

*p≤.05
**p≤.01
*** p≤.001

© 2024 Zhu Y et al. *JAMA Network Open.*
