# Supplementary material for: Differential Associations of Alcohol Use With Ischemic Heart Disease Mortality by Socioeconomic Status in the US, 1997-2018
Source: JAMA Netw Open. 2024 Feb 1;7(2):e2354270. doi: 10.1001/jamanetworkopen.2023.54270 (PMC10835511; doi:10.1001/jamanetworkopen.2023.54270)
Supplement: Supplement 2. — Data Sharing Statement [file jamanetwopen-e2354270-s002.pdf]

Data Sharing Statement

Zhu. Differential Associations of Alcohol Use With Ischemic Heart Disease Mortality by Socioeconomic Status in the US, 1997-2018. *JAMA Netw Open*. Published February 01, 2024. doi:10.1001/jamanetworkopen.2023.54270

Data
Data available: No

Additional Information
Explanation for why data not available: The data of this study is for restricted use only at the NCHS Research Data Center and cannot be made publicly available.
